# Increasing the Content of Bioactive Compounds in Apple Juice Through Direct Ultrasound-Assisted Extraction from Bilberry Pomace

**DOI:** 10.3390/foods13244144

**Published:** 2024-12-21

**Authors:** Violeta Nour

**Affiliations:** Department of Horticulture & Food Science, University of Craiova, 13 AI Cuza Street, 200585 Craiova, Romania; vionor@yahoo.com

**Keywords:** bilberry pomace, apple juice, ultrasound-assisted extraction, anthocyanins, phenolic compounds, antioxidant activity, optimization

## Abstract

The increasing trend of diet-related chronic diseases has stimulated research into developing new food products and beverages with health-promoting potential. At the same time, new resources, including plant by-products, are currently being investigated as a sustainable source of bioactive compounds. In this context, the present study focused on the enrichment of apple juice with anthocyanins and other phenolic compounds by direct ultrasound-assisted extraction (UAE) from bilberry pomace. Response surface methodology combined with a Box–Behnken design was used to find the optimal extraction conditions for maximizing the total anthocyanin content (TAC), total phenolic content (TPC) and DPPH radical scavenging activity (RSA) in the enriched apple juices and to characterize their phenolic profile as influenced by the extraction temperature. UAE from 15% bilberry pomace during 15 min in apple juice at 80 °C resulted in the highest TAC (262.73 mg CGE/L), TPC (1700.91 mg GAE/L) and RSA (8.93 mmol Trolox/L) of the enriched apple juice. The chromatographic polyphenolic profile of the control and enriched juices showed that, besides anthocyanins, phenolic acids (chlorogenic, gallic, caffeic, 3-hydroxybenzoic, p-coumaric, ellagic and protocatechuic acids) and flavonoids (epigallocatechin and catechin) were extracted from the bilberry pomace directly in the apple juice, while the extraction temperature differently impacted the content of individual phenolic compounds.

## 1. Introduction

According to FAOSTAT (2022), 96 million tons per year of apples were produced worldwide, apple being one of the most consumed fruits all over the world [1]. Most of the global apple production is consumed as a fresh fruit while 25–30% is processed, apple juice being the second most popular juice worldwide [2]. Apples are considered a good source of phenolic compounds, consisting mainly of flavan-3-ols (especially proanthocyanidins) (~50%), dihydrochalcones (phloridzin and phloretin), phenolic acids, flavonols and small amounts of anthocyanins [3,4]. However, several studies have reported that apples showed intermediate or even low phenolic content as compared with other fruits [5,6]. Moreover, apple polyphenols are localized mainly in the peels and are poorly extracted into the juice [7]. Considering that juices are a more and more popular way of consuming fruits [8], and with a view to developing new beverages with a high positive impact on public health, the industry is currently focusing on exploring new plant resources, including plant by-products, that may be used to enhance the juices ‘content of bioactive compounds [9,10,11]. Due to its wide availability and popularity, apple juice may be considered as an ideal candidate for enrichment with phenolic compounds in order to increase its functionality [12]. Given this background, the present study focused on the enrichment of apple juice with anthocyanins and other phenolic compounds through the extraction of bilberry pomace directly in the apple juice. To our knowledge, there are only few works on the enrichment of juices through direct extraction from fruit by-products. For instance, Altunkaya et al. [13] explored pomegranate peel extract to fortify apple juice in the 0.5 to 2.0% (*w/w*) concentration range, while Kolniak-Ostek et al. [14] obtained functional beverages, with increased phytochemical content, by the addition of 0.5%, 1% and 5% apple leaves to cloudy apple juices.

Bilberry (*Vaccinium myrtillus* L.), one of the most widespread wild berries in northern Europe, is an extremely rich natural source of anthocyanins, which impart the dark blue and purple color to the fruit [15,16]. Some evidence suggests that the intake of bilberry as fruits or juices may interfere with cancer progression by preventing the growth of cancer cells and inducing apoptosis [17,18,19,20], may protect proteins, lipids and DNA against oxidative deterioration [21,22] and may help to prevent and treat various chronic inflammatory disorders by reducing the levels of the inflammatory markers [23,24]. The involvement of bilberry in the prevention and treatment of macular degeneration, diabetes, hepatic injury, Alzheimer’s disease and dementia was also reported [25,26]. The health benefits of bilberries have been related to the remarkable content of bioactive compounds including anthocyanins, phenolic acids, flavanols, flavonols, coumarins, procyanidins, tannins, terpenoids and stilbenes [23,25]. However, anthocyanins have been shown to be the compounds with the highest antiproliferative effects in bilberry. Anthocyanins are reported to exhibit health-promoting effects including anti-diabetic, anti-carcinogenic, antimutagenic, anti-obesity and antihypertensive effects owing to their powerful anti-inflammatory, antithrombotic and antioxidant activities [27]. Delphinidin is the main aglycone in bilberry fruit, followed by cyanidin, malvidin petunidin and peonidin, while the most frequent sugars bound to these aglycones are glucose, galactose and arabinose [28].

Due to their limited shelf life, wild bilberries are often processed in juices, jams, jellies and wines. The solid residues obtained after juice processing, called pomace, consisting of skins, seeds and residual pulp and accounting for approximately 20–30% of the fruit [29], are an abundant source of anthocyanins and flavonoids. Several previous studies demonstrated that the bioactive content and antioxidant activity in these by-products are several times higher compared to the fruit juice or the whole fruit [30,31,32]. However, the pomace is inefficiently used as animal feed or fertilizer, but most often it is discarded into soil and landfills as waste, generating severe environmental problems, especially considering its low pH [33]. Therefore, achieving a better exploitation of these by-products can make a contribution to the mitigation of pollution and to sustainable development [34]. The high moisture content of the pomace makes it very prone to microbial spoilage; therefore, in most cases, the pomace requires drying. Subsequently, the resulting powders or their extracts can be used to produce natural colorants, nutraceuticals, functional foods, pharmaceuticals and cosmetics [32,35,36].

Various extraction methods have been developed to efficiently recover bioactive compounds from berry pomaces in the last years, including conventional solid–liquid extraction techniques, microwave-assisted, ultrasound-assisted, supercritical CO_2_, pressurized liquid and enzyme-assisted extractions [33,37,38]. Ultrasound-assisted extraction (UAE) is considered a “green” or an “environmentally friendly” extraction method compared to classical maceration because it reduces energy consumption, permits the use of alternative solvents and leads to obtaining a safe, high-yield and high-quality extract [37,39,40]. Sonication time, temperature, solvent composition, solid/solvent ratio, particle size and matrix composition, and the power and frequency of ultrasonic waves are factors affecting the yield, composition and properties of the extracts [41]. Therefore, it is difficult to generalize the optimum UAE operating conditions for the extraction of specific bioactive compounds from various matrices. Since the high cost of drying creates additional limitations to pomace valorization, the present study proposes the extraction of bioactive compounds from the fresh bilberry pomace. Furthermore, to avoid removing the solvent used in the extraction in view of the subsequent incorporation of the extracted bioactive compounds into food products, the present study proposes the use of apple juice as a solvent for the direct extraction of anthocyanins and other phenolic compounds from bilberry pomace. Although some previous studies have reported on the extraction of phenolic compounds from bilberry pomace, the direct extraction of bilberry pomace in apple juice and the phenolic composition and antioxidant activity of the enriched juices have not been studied.

The aim of the present study was to enrich apple juice with anthocyanins and other phenolic compounds by direct ultrasound-assisted extraction from bilberry pomace. Therefore, our objectives were (i) to find the optimal extraction conditions (temperature, extraction time and solid percent) for maximizing the total anthocyanin content, total phenolic content and DPPH radical scavenging activity in the enriched apple juice; and (ii) to characterize the phenolic composition of the enriched apple juices as compared to the control and to establish the effects of temperature on the extraction of phenolic compounds from bilberry pomace in apple juice.

## 2. Materials and Methods

### 2.1. Plant Materials

The bilberry pomace used in this study was the by-product that remained after the mechanical pressing of bilberry juice, consisting of peels, seeds and residual pulp. Bilberry (*Vaccinium myrtillus* L.) fruits were harvested in August 2023 at optimum fruit maturity from the spontaneous flora of the hilly area of Vâlcea county (South-West Oltenia Region, Romania). Two batches of fresh bilberry pomace (2 kg) were collected on different days during bilberry juice production without enzymatic treatment from Jiancom S.R.L., an industrial fruit processing company in Vaideeni (Vâlcea county, South-West Romania, Romania). They were transported to the lab, frozen at −18 °C and thawed at 5 °C prior to the experiments. Commercial apple juice from Granini (Mâcon, France) (total carbohydrates 10.00 g/100 mL, of which sugars 9.7 g/100 mL) was the chosen solvent for the direct extraction of bilberry pomace. The total soluble solids content of the juice was determined using a digital refractometer (Hanna Instruments, Woonsocket, RI, USA) and expressed as a percentage. Juice titratable acidity was determined by titration to pH 8.2 with 0.1 N NaOH solution and the results were expressed as grams of malic acid per liter.

### 2.2. Chemicals

Folin–Ciocalteu reagent (2 N), potassium chloride, 6-hydroxy-2,5,7,8-tetramethylchroman-2-carboxylic acid (Trolox, 98% purity) and hydrochloric acid (37%) were purchased from Merk (Darmstadt, Germany). Methanol (MeOH) was of HPLC grade and was obtained from Aladdin (Shanghai, China). Gallic acid (99% purity), anhydrous sodium carbonate (99% purity), anhydrous sodium acetate (98% purity) and 2,2-diphenyl-1-picrylhydrazyl (DPPH, 90% purity) were from Sigma-Aldrich (Steinheim, Germany). The polyphenol standards syringic acid (98%), ellagic acid (95%), 4-hydroxy-3-methoxy-cinnamic acid (95%), 3-hydroxybenzoic acid (95%), caffeic acid (95%), protocatechuic acid (96%), vanillic acid (95%), coumaric acid (98%), catechin (95%), epicatechin (96%), epigallocatechin (97%), quercetin (95%), rutin (95%) and resveratrol (99%) were purchased from Sigma (Sigma-Aldrich GmbH, Steinheim, Germany) while chlorogenic acid (95%) and ferulic acid (97%) were from the European Pharmacopoeia (Strasbourg, France) ).

### 2.3. Proximate Composition of Bilberry Pomace

The moisture content was determined after drying the fresh bilberry pomace to a constant weight at 103 °C in a Memmert ULM500 drying oven (Uden, The Netherlands). The crude protein, crude fiber, fat and ash contents of the fresh bilberry pomace were analyzed following AOAC standard methods. The protein content was measured according to the Kjeldahl method by using an automated nitrogen analyzer (UDK 149, Velp Scientific, Milan, Italy) while the fat content was determined after extraction in organic solvents by using a Soxhlet automatic extraction system (SER 148/3, Velp Scientific, Usmate, Italy). The crude fiber content was estimated using an automatic analyzer (Fibertec 2010, Tecator, Sweden) after digestion with acid and alkali, and the ash content was quantified as the sample residue after incineration at 550 °C in a Caloris CL 1206 oven (Bucharest, Romania). Triplicate samples were analyzed and the average values were reported. The determinations were completed in triplicate, and values are expressed on a dry weight basis as means ± standard deviations.

### 2.4. Experimental Design

Response surface methodology (RSM) was applied for modeling and optimizing the extraction of anthocyanins and polyphenols from the bilberry pomace in the apple juice.

The main objective of RSM is to optimize the response surface of an interest variable by quantifying the relationship between some controllable process parameters and the obtained response surface. A series of experiments is designed and the responses of the interest variables are recorded. Based on the responses, a mathematical model of the second-order response surface is developed with the best fittings. The model allows us to find the optimal set of process parameters that determine the best (maximum or minimum) value of the response variable and to plot in two or three dimensions the effects of the process parameters on the response.

A second-order model utilized in response surface methodology is presented in the following polynomial equation:

$$Y=\beta_{0}+\sum_{i=1}^{k}\beta_{i}x_{i}+\sum_{i=1}^{k}\beta_{ii}x_{i}^{2}+\sum_{i=1}^{k-1}\sum_{j=2}^{k}\beta_{ij}x_{i}x_{j}$$

where *Y* is the response, *x*_1_, *x*_2_, …, *x_k_* are the process parameters, *β*_0_ is the model constant coefficient, and *β_i_* is the linear coefficient, *β_ii_* is the quadratic coefficient and *β_ij_* is the interaction coefficient of variables *i* and *j*.

A Box–Behnken design with three factors and three levels was used to design a second-order polynomial model between the response variables and the process parameters and to optimize the extraction process. Temperature (*x*_1_, degrees Celsius), extraction time (*x*_2_, minutes) and solid (bilberry pomace) percent (*x*_3_, %) were the controllable process parameters (independent variables) studied, while total anthocyanin content, total phenolic content and DPPH radical scavenging activity in the apple juice after the direct extraction of bilberry pomace were chosen as the response variables. The coded and actual levels of the process parameters used in the experiment are given in Table 1.

The adequacy of the model was assessed based on the analysis of variance (ANOVA), which provides numerical information on the *F* ratio and *P* value for the model parameters as well as the R-Squared (*R*^2^) value of the model, indicating the extent to which the model as fitted explains the variability of the response. The statistical significance of the model and model parameters were determined at 95% confidence level.

Response surface plots were generated to evaluate response parameters (*Y*_1_ = TAC, *Y*_2_ = TPC and *Y*_3_ = RSA) by holding constant one process parameter (at its medium level) and plotting the response against the other two parameters.

### 2.5. Extraction of Bilberry Pomace in Apple Juice

Optimization of the extraction was performed using process parameters that could be applied in the fruit juice processing industry, with apple juice as the extraction solvent. After thawing, the bilberry pomace was accurately weighed in a 50 mL flask and mixed with apple juice to reach the appropriate solid percent (5%, 10% and 15% *w/w*). The flasks were shaken for one minute and then immersed in an ultrasonic bath (Bandelin Sonorex Digital 10P, Bandelin Electronic GmbH, Berlin, Germany, 35 kHz, 480 W) either for 30, 60 or 90 min at 20, 50 or 80 °C, according to the Box–Behnken experimental design presented in Table 2. Water at the appropriate temperature was poured into the ultrasound bath and subsequently the temperature was controlled and maintained at the desired extraction temperature (± 2 °C) by replacing the water in the bath with cold or heated water. Immediately after each treatment, the juice was separated from the residue by filtration through filter paper (Whatman nr. 1). Each extraction was replicated two times and each replicate extract was analyzed in triplicate for total anthocyanin content, total phenolic content and DPPH radical scavenging activity.

### 2.6. Total Anthocyanin Content

The total anthocyanin content was quantified according to the pH differential method presented by Giusti and Wrolstad [42]. Firstly, the juice was diluted five times with distilled water as the absorbance at 510 nm should fall below 1.4 AU. Afterwards, the diluted juice was then diluted 2:25 (*v/v*) in 0.025 M potassium chloride buffer (pH 1.0) and 0.4 M sodium acetate buffer (pH 4.5). After 15 min of incubation in the dark at 25 °C, the absorbance was measured at 510 and 700 nm using a Varian Cary 50 UV spectrophotometer (Varian Co., Palo Alto, CA, USA).

The total anthocyanin content of the juices was calculated as mg cyanidin 3-*O*-glucoside equivalents (CGE)/L using the formula below:

Total anthocyanin content (mg CGE/L) = (ΔA × M × DF × 1000)/(ε × l)

where ΔA = (A_510nm_ − A_700nm_)_pH 1.0_ − (A_510nm_ − A_700nm_)_pH 4.5_; M (molecular mass) = 449.2 g/mol; DF (dilution factor) = 31.25; ε (molar extinction coefficient) = 29,600 L/(mol·cm); l (cuvette path length) = 1 cm; and 1000 = conversion factor from g to mg.

### 2.7. Total Phenolic Content

To determine the total phenolic content, the juices were extracted with 80% (*v/v*) aqueous methanol. The total phenolic content was determined by the Folin–Ciocalteu method according to Singleton et al. [43]. Briefly, an aliquot of juice extract (0.1 mL) was mixed with 5 mL of distilled water and 0.5 mL of Folin–Ciocalteu reagent (diluted 1:1 with distilled water). After 3 min, 1.5 mL of sodium carbonate solution (20%, *w/v*) was added, and the mixture was finally diluted with 2.9 mL of distilled water. After stirring and keeping at 40 °C in the dark for 30 min, the absorbance was measured at 765 nm with a Varian Cary 50 UV spectrophotometer (Varian Co., Palo Alto, CA, USA). The results were expressed as milligrams of gallic acid equivalents per liter of juice (mg GAE/L) based on a calibration curve of gallic acid standards (*y* = 0.0011*x* − 0.0272, *R*^2^ = 0.9988).

### 2.8. DPPH Radical Scavenging Activity

The antioxidant activity of the juices was evaluated as DPPH free radical scavenging activity assessed using the 2,2-diphenyl-1-picrylhydrazyl (DPPH) method [44]. Briefly, 3 mL of DPPH methanolic solution (0.004%) were added to 50 μL of juice extract. The mixture was shaken vigorously and kept in the dark for 30 min at room temperature (22 °C). After incubation, the absorbance of the mixed solution was read at 517 nm using a Varian Cary 50 UV–VIS spectrophotometer (Varian Co., Palo Alto, CA, USA). A control sample was prepared in the same way, except that 50 μL of pure methanol was taken instead of juice extract. The inhibition percentage of the DPPH radical by the sample was calculated according to the following formula:

DPPH scavenging activity (%) = [1 − absorbance of the sample/absorbance of the control] × 100.

An analytical curve was plotted using Trolox (6-hydroxy-2,5,7,8-tetramethylchroman-2-carboxylic acid) as reference standard (*y* = 31.428*x*, *R*^2^ = 0.9964), and the results were expressed as mmol Trolox per liter of juice.

### 2.9. Individual Polyphenolic Compounds

The polyphenolic profile was determined by liquid chromatography of the apple juice (AJ) as well as of AJBP20, AJBP50 and AJBP80 juices obtained through the direct ultrasound-assisted extraction of 10% bilberry pomace in apple juice during 30 min at 20 °C, 50 °C and 80 °C, respectively.

In order to determine the individual phenolic compounds, the extraction was conducted according to Saracila et al. [45]: 1 mL of juice was extracted in 9 mL of water/methanol/acetic acid (69:30:1, *v*/*v*/*v*) in a screw-cap test tube. After incubation at 50 °C for 60 min in a shaking water bath (Memmert, Schwabach, Germany), the extract was filtered through a 0.45 μm filter.

The individual phenolic compounds were quantified by RP-HPLC-DAD on a Vanquish Core HPLC system (Thermo Fisher Scientific, Bremen, Germany) equipped with a photodiode array detector [45]. Chromatograms were recorded at 254, 270, 280, 310 and 320 nm. Separation was performed on a BDS HyperSil C18 column (250 × 4 mm, 5 µm particle size) from Thermo Fisher Scientific (Bremen, Germany). Three mobile phases, (A) acetic acid (1%) in distilled water (*v*/*v*), (B) methanol and (C) acetonitrile, were used at a flow rate of 0.5 mL/min, with the following binary gradient profile: 0–15 min—5% (B), 5% (C); 15–20 min—4% (B), 15% (C); 20–25 min—3% (B), 25% (C); 25–40 min—2% (B), 38% (C); 40–50 min—5% (B), 5% (C). The injection volume was 40 μL and the column temperature was set at 25 °C. The contents of phenolic compounds were determined based on the calibration curves of individual standards.

### 2.10. Statistical Analysis

RSM was performed using Statgraphics Centurion XVI.I software (StatPoint Technologies, Warrenton, VA, USA). Pearson correlations were performed to assess the relationships between the total anthocyanin content, total phenolic content and DPPH radical scavenging activity using Statgraphics Centurion XVI software (StatPoint Technologies, Warrenton, VA, USA). The results of the chromatographic analysis were presented as mean values ± standard deviation of the triplicate determinations. The results were compared using one-way analysis of variance (ANOVA) followed by the LSD test and differences at *p* < 0.05 were considered as statistically significant.

## 3. Results

The apple juice used in the present study had soluble solid content = 11.5%, pH = 3.7 and titratable acidity (in terms of malic acid) = 5.7 g/L. The moisture content of the fresh bilberry pomace was 78.92 ± 0.18%. The results of the proximate composition of the bilberry pomace expressed on a dry weight basis (dw) were as follows: crude protein—8.17 ± 0.27 g/100 g dw, crude fat—8.28 ± 0.23%, crude fiber—11.54 ± 0.32 g/100 g dw and ash—1.15 ± 0.22 g/100 g dw. A total phenolic content of 37.75 ± 0.62 mg GAE/g dw and a total anthocyanin content of 28.75 ± 0.57 mg CGE/g dw were found in the bilberry pomace used in the experiment.

### 3.1. Optimization of Extraction Process

In the present study, the direct ultrasound-assisted extraction of bilberry pomace in apple juice was optimized by response surface methodology to obtain the maximum total anthocyanin content, total phenolic content and DPPH radical scavenging activity in the enriched juices. Many previous studies demonstrated that ultrasound increases the extraction yield (a) by the cavitation effects of UAE and the disruption of the cellular matrix, thus allowing a greater accessibility of the solvent to the internal structure of the matrix and (b) by intensifying the washing out of the extracts from the matrix due to the higher contact surface area between the solid and the liquid phases [39,46]. Based on these considerations, UAE was chosen in the present study as a “green technique” to efficiently recover anthocyanins and other phenolic compounds from the bilberry pomace using apple juice as a clean “green” solvent. Temperature, extraction time and solid (bilberry pomace) percent were the process parameters optimized in the experiment using a Box–Behnken design combined with RSM. The mean experimental data for TAC, TPC and RSA obtained from a 15-run experiment with two replicates are presented in Table 2.

The coefficients of the predicted quadratic models for the responses and the analysis of variance of the models for TAC, TPC and RSA are presented in Table 3. Positive values of the regression coefficients indicate synergistic effects, whereas negative values are indicative of antagonistic effects. The regression analysis showed that the mathematical models generated for TAC, TPC and RSA do not have significant lack-of-fit values (>0.05), so they appear to be appropriate to predict the responses and to describe the relationship between the independent variables at a 95.0% confidence level within the studied variables range. The R-squared statistic shows the percentage of variation in the response that has been explained by the fitted model. The R-squared values for the three models range from 97.36% to 99.86%. 3D response surface plots are generated to graphically represent the interaction of two independent variables and their influence on the selected response.

### 3.2. Central Composite Design and Surface Plots for Total Anthocyanin Content

**Figure 1:**
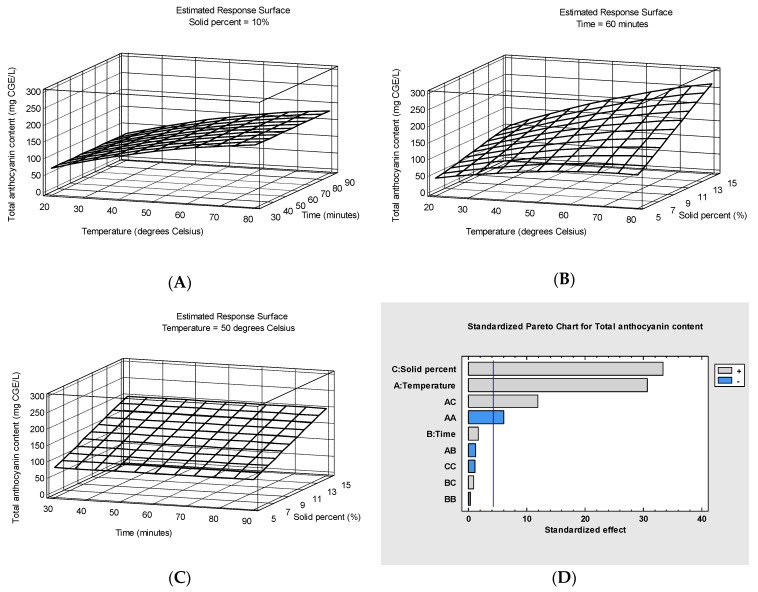
Response surface plots of total anthocyanin content (mg CGE/L) as a function of temperature and extraction time at 10% solid percent (**A**), temperature and solid percent at 60 min extraction time (**B**) and extraction time and solid percent at 50 degrees Celsius temperature (**C**) and Pareto chart for total anthocyanin content (**D**).

The model for TAC shows that only four effects have *p*-values less than 0.05, indicating that they have a significant impact on the response at the 95.0% confidence level: solid percent, temperature, solid percent × temperature and solid percent × solid percent. Figure 1 presents the response surface plots of total anthocyanin content (mg CGE/L) as a function of temperature and extraction time at 10% solid percent (A), temperature and solid percent at 60 min extraction time (B) and extraction time and solid percent at 50 degrees Celsius temperature (C). The dependency obtained after excluding the insignificant effects (*p*-values higher than 0.05) is presented by means of the following equation, which can be used to predict the extraction efficiency of total anthocyanins:

$$Y_{1}=1.52899x_{1}+1.87342x_{3}+0.192067x_{1}x_{3}-0.01689x_{1}^{2}-9.3611$$

where *Y*_1_ is the total anthocyanin content (mg CGE/L), *x*_1_ is the temperature (°C) and *x*_3_ is the solid percent (%).

The *p*-value for lack-of-fit in the ANOVA table was 0.9292, which is higher than 0.05, so the model appears to be adequate for the observed data at the 95.0% confidence level. The model reveals that there is a strong positive correlation between the total anthocyanin content extracted in the apple juice from the bilberry pomace and the extraction time, temperature and their interaction.

The combination of factor levels that maximizes TAC (predicted value = 263.74 mg CGE/L) is 80 °C temperature, 60 min extraction time and 15% solid percent. Increasing the temperature accelerates extraction by enhancing the mass transfer rate and molecular diffusion but it also accelerates the degradation of heat-sensitive bioactive compounds. In a study on the effect of different extraction temperatures (22, 40, 60, 80 and 100 °C) in combination with different extraction times (4, 15, 30 and 45 min) on the aqueous extraction of anthocyanins and phenolic compounds from bilberry press residue, Aaby et al. [47] also found that temperature, but not extraction time, had a significant positive effect on the total anthocyanin content of the extract. In good agreement with our results, they found that the highest TAC values were achieved in the extracts obtained at 80 °C. The TAC values in the enriched apple juices were found in the range of 41.73–262.73 mg CGE/L of juice (solid percent between 5 and 15%) while Aaby et al. [47] reported TAC values between 95 and 625 mg CGE/L of aqueous extract made from 5 g of bilberry press residue and 15 mL of water. In contrast, Bamba et al. [41] reported that the extraction duration significantly affected the ultrasound-assisted recovery of anthocyanins from blueberry pomace, TAC after 90 min extraction being significantly higher than after 30 or 60 min. Ćujić et al. [48] also reported greater TAC in the extracts of dried chokeberries in 50% ethanol after 60 min UAE than after 30 min while Hu et al. [49] found that the amount of TAC recovered by UAE from freeze-dried blueberry pomace achieved saturation at 10–30 min, and decreased significantly thereafter, regardless of the solvent used. Moreover, Lapornik et al. [50] reported that longer extraction times (1–24 h) of redcurrant and blackcurrant by-products in water without sonication can lead to a decrease in TPC and TAC, indicating that a long extraction in water may cause the degradation of some bioactive compounds.

### 3.3. Central Composite Design and Surface Plots for Total Phenolic Content

**Figure 2:**
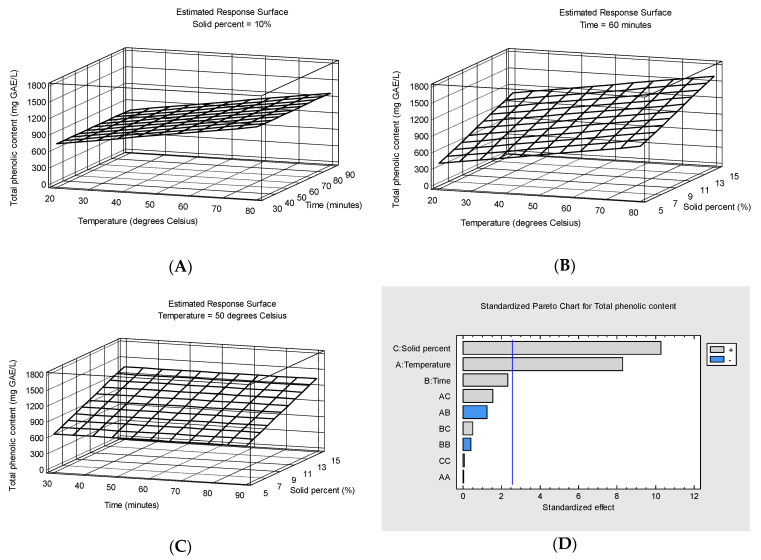
Response surface plots of total phenolic content (mg GAE/L) as a function of temperature and extraction time at 10% solid percent (**A**), temperature and solid percent at 60 min extraction time (**B**) and extraction time and solid percent at 50 degrees Celsius temperature (**C**) and Pareto chart for total phenolic content (**D**).

Figure 2A–C show the response surface plots of total phenolic content (mg GAE/L) as a function of temperature and extraction time at 10% solid percent (A), temperature and solid percent at 60 min extraction time (B) and extraction time and solid percent at 50 degrees Celsius temperature (C). The Pareto chart for total phenolic content shows that only solid percent and temperature have a significant impact on the response at the 95.0% confidence level. The variation obtained after excluding the insignificant effects (*p*-values higher than 0.05) is presented by means of the following equation:

$$Y_{2}=8.86367x_{1}+66.023x_{3}-101.292$$

where *Y*_2_ is the total phenolic content (mg GAE/L), *x*_1_ is the temperature (°C) and *x*_3_ is the solid percent (%).

The *p*-value for lack-of-fit was 0.7351, which is above 0.05, so the model appears to be appropriate for the observed data at the 95.0% confidence level.

The temperature and solid percent had a significant positive correlation with TPC while the influence of the extraction time is not significant in the range 30–90 min. Ćujić et al. [48] also reported no difference between the TPC extracted after 30 and 60 min from dried chokeberry using maceration. The extraction conditions that maximize total phenolic content (predicted value = 1698.82 mg GAE/L) were 80 °C temperature, 90 min extraction time and 15% solid percent.

Previously, Aaby et al. [47] found the highest total phenolic content in the aqueous extracts of bilberry press residue was obtained at 100 °C and 15 min extraction time, not significantly higher than in the extracts obtained at 80 °C and 15 min or 30 min extraction time. In good agreement with our results, TPC in the extracts ranged from 249 to 1153 mg GAE/L of extract [47].

### 3.4. Central Composite Design and Surface Plots for Radical Scavenging Activity

**Figure 3:**
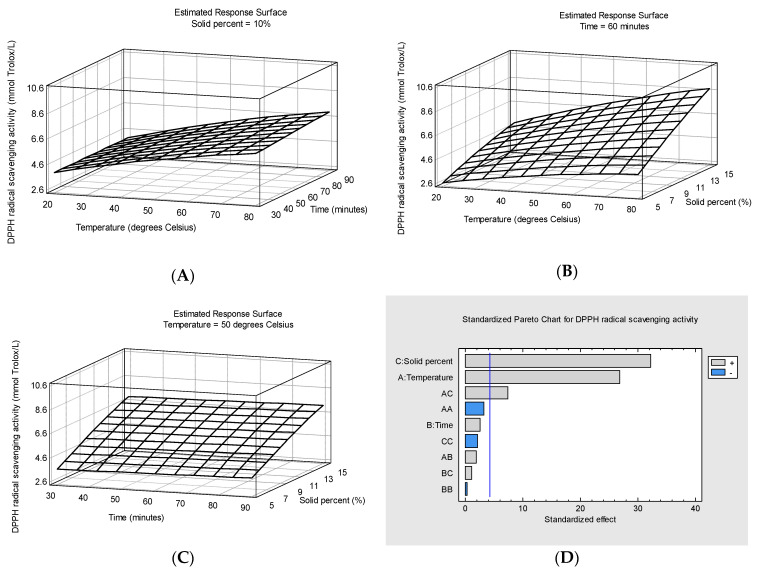
Response surface plots of DPPH radical scavenging activity (mmol Trolox/L) as a function of temperature and extraction time at 10% solid percent (**A**), temperature and solid percent at 60 min extraction time (**B**) and extraction time and solid percent at 50 degrees Celsius temperature (**C**) and Pareto chart for DPPH radical scavenging activity (**D**).

Figure 3A–C present the response surface plots for radical scavenging activity values, as measured by the DPPH scavenging capacity assay, expressed as mmol Trolox/L.

The Pareto chart (Figure 3D) indicates that only two linear coefficients (solid percent and temperature) and their interaction (solid percent × temperature) were significant for the radical scavenging activity of the extracts while the extraction time and the other coefficients had an insignificant effect. The following equation describes the variation in RSA after excluding the insignificant effects (*p*-values higher than 0.05):

$$y=0.01x_{1}+0.15175x_{3}+0.0036x_{1}x_{3}+1.53983$$

where Y is the radical scavenging activity (mmol Trolox/L), *x*_1_ is the temperature (°C) and *x*_3_ is the solid percent (%).

The solid percent and temperature as well as their interaction showed a positive impact on the increase in antioxidant activity in all the enriched juice samples. In contrast, in a study on the optimization of bilberry pomace enzyme-assisted extraction, Syrpas et al. [38] found that temperature in the tested range (30–50 °C) did not significantly influence the antioxidant activity of the extracts, as temperature was not a statistically significant model term, while enzyme concentration, pH and time were significant in determining the radical scavenging activity of the water-soluble fractions of bilberry pomace after enzyme-assisted extraction [51]. The maximum predicted DPPH radical scavenging activity (9.063 mmol Trolox/L) was obtained under the following estimated optimum conditions: temperature = 79.6 °C, extraction time = 89.97 min and solid percent = 15%.

### 3.5. Simultaneous Response Optimization

Considering all the observed responses, the extraction of bilberry pomace in apple juice was optimized with the aim of maximizing the three responses (TPC, TAC and RSA) based on the desirability function within the selected range of the independent variables.

Based both on the observed and predicted responses, the most desirable results were obtained for 80 °C temperature, 60 min extraction time and 15% solid percent. The overall desirability of the model was 0.986. Under these conditions, an enriched apple juice with a total anthocyanin content of 262.73 mg CGE/L, a total phenolic content of 1700.91 mg GAE/L and a radical scavenging activity of 8.93608 mmol Trolox/L was obtained. These values were in agreement with the model prediction. He et al. [39], in a study on the optimization of UAE of total anthocyanins and total phenolics from blueberry wine pomace using RSM and a Box–Behnken design, found that the optimized conditions were 61.03 °C for the extraction temperature and 23.67 min for the sonication time, while dos Santos et al. [52] found that UAE for 45 min was optimum to extract bioactive compounds from blueberry and raspberry pomace. Piechowiak et al. [32] showed that the highest content of polyphenols was extracted from blueberry fruit waste after 15 min of the process, at 80 °C using 50% acidified ethanol.

The results for RSA in all the enriched juices were strongly positively correlated with TAC and TPC and the correlations were statistically significant (*p* < 0.05) (Table 4).

Radical scavenging activity in plant products is the result of the cumulative antioxidant action of different compounds including polyphenols and vitamins (ascorbic acid) as well as of their synergic and/or antagonistic interactions. The present results confirm the strong relationship between the antioxidant activity and the concentration of polyphenols and the major contribution of the anthocyanins in the enriched juices. Similar positive correlations between antioxidant activity and TPC have been reported previously in apple juice enriched with various fruits, berry extract and flower by-products [8,53]. Previous studies found that ultrasound treatment determined a significant increase in the level of ascorbic acid in apple juice [54] as well as in other fruit juices [55] and attributed this behavior to the better extractability of ascorbic acid and to the elimination of the dissolved oxygen during cavitation. Other studies found that ultrasound treatments were more effective in preserving ascorbic acid, bioactive compounds and antioxidant capacity than thermal treatments [56,57,58]. The increase in the antioxidant activity during sonication could be attributed also to the formation of degraded polysaccharides having better antioxidant properties than ordinary polysaccharides in combination with phenolic compounds [59]. However, Araújo et al. [60] found that increased ultrasound intensity could generate hydroxyl radicals involved in the decomposition of bioactive compounds. Recently, Lepaus et al. [61] concluded that the effects of ultrasound treatment on the bioactive compounds depend on the ultrasound intensity and food matrix. They noticed that the observed increase in the bioactive content after the ultrasound treatment could be the result of the better release of the compounds from their compartments facilitated by cavitation during chemical analysis; therefore, they recommended that future investigations should focus on the bioavailability of the bioactive compounds from ultrasonically treated food matrices.

Recent studies reported on the structural and chemical damage to pectin in ultrasonically processed fruit juices [59,61,62], consisting of pectin defragmentation and a reduction in the degree of methylation due to cavitation effects. These types of damage determine the reduction in viscosity and the delay in the sedimentation index during juice storage. Other effects of ultrasound reported in fruit juices are the denaturation of pectin methyl esterase accounting for pectin hydrolysis and the increase in the fibers’ hydration capacity. Considering the high pectin content of bilberry pomace [34], a future study should analyze the transformations of pectin and its interaction with the phenolic compounds in the juices resulting from the direct extraction of bilberry pomace in apple juice as well as the effects on their quality, bioactivity and stability.

### 3.6. Chromatographic Phenolic Profile of Control and Enriched Apple Juices

**Figure 4:**
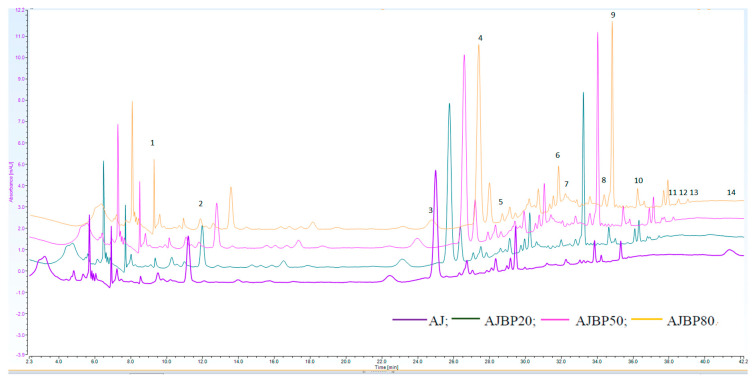
Representative HPLC-DAD chromatograms of phenolic compounds in apple juice (AJ) and in apple juices enriched with bioactive compounds through direct ultrasound-assisted extraction from bilberry pomace at 20 °C (AJBP20), 50 °C (AJBP50) and 80 °C (AJBP80) (λ = 280 nm; extraction time = 30 min, solid percent = 10%). Peak identification: (1) gallic acid; (2) epigallocatechin; (3) catechin; (4) chlorogenic acid; (5) caffeic acid; (6) hydroxybenzoic acid; (7) cyanidin 3-glucoside; (8) p-coumaric acid; (9) ellagic acid; (10) ferulic acid; (11) protocatechuic acid; (12) resveratrol; (13) quercetin; (14) kaempferol.

The results for the content of polyphenolic compounds determined by HPLC-DAD in the control apple juice and in the apple juices enriched with bioactive compounds through direct ultrasound-assisted extraction from bilberry pomace at 20, 50 and 80 °C are presented in Table 5 while representative HPLC-DAD chromatograms are shown in Figure 4.

The total phenolic content of the apple juice, determined as the sum of the individual phenolic compounds analyzed by HPLC, was 284.7 mg/L (Table 5). The most abundant phenolic acid was chlorogenic acid (136.22 mg/L) followed by gallic acid (23.37 mg/L) and caffeic acid (4.04 mg/L) while p-coumaric was not detected. Among flavonoids, catechin was found in the largest quantity (54.59 mg/L), followed by epigallocatechin (33.30 mg/L). In good agreement with our results, Eisele and Drake [63] reported 70.7 ± 79.3 mg/L chlorogenic acid, 4.9 ± 4.6 mg/L caffeic acid and 16.6 ± 25.6 mg/L epigallocatechin in apple juice prepared from 175 apple varieties. In contrast to our data, Eisele and Drake [63] found only 1.2 ± 6.1 mg/L catechin and 4.9 ± 3.3 mg/L p-coumaric acid while Kahle et al. [64] found only 52.9–95.6 mg/L chlorogenic acid, 3.9–4.4 mg/L caffeic acid and 14–19 mg/L epicatechin in commercial clear apple juices. The phenolic profile of the apple juice in the present study was also in accordance with previous findings [65]. Tian et al. [66] reported similar contents of the phenolic constituents in apple juices made from sixteen apple cultivars: 79.7–313.7 mg chlorogenic acid/L, 2.3–6.1 mg gallic acid/L, 0–9.4 mg protocatechuic acid/L, 0–3.7 mg/L caffeic acid, 13.3–33.3 mg/l epigallocatechin and 2.3–19.3 mg/L catechin. Maragò et al. [67] also found similar chlorogenic acid contents (103.9 mg/L and 166.2 mg/L) but higher catechin contents (89.1 and 148.6 mg/L) in Golden Delicious and Panaia-red apple juices.

For most phenolic compounds, the direct extraction of bilberry pomace in apple juice resulted in significant increases in their content in the resulting juices. Chlorogenic, gallic, caffeic, 3-hydroxybenzoic, p-coumaric, ellagic and protocatechuic acids are the phenolic acids extracted from bilberry pomace directly in apple juice. Previously, Piechowiak et al. [32] showed that blueberry pomace extract is a good source of anthocyanins, flavanols and proanthocyanidins and flavonols, as well as chlorogenic acid. Čanadanović-Brunet et al. [25] previously quantified protocatechuic, gallic, ferulic and ellagic acids in the wild bilberry pomace extracts made in 80% methanol containing 0.05% acetic acid, while Vázquez-Vázquez et al. [68] found predominantly syringic, chlorogenic and caffeic acids from the group of hydroxycinnamic acids and gallic acid from the group of hydroxybenzoic acids in the blueberry pomace extract.

In the case of epigallocatechin, catechin, gallic, caffeic and ellagic acids, the higher the extraction temperature was, the greater the increase in their content in the enriched apple juice. However, the content of chlorogenic, protocatechuic, 3-hydroxybenzoic, p-coumaric and ferulic acids extracted from the bilberry pomace decreased at higher temperature (80 °C), probably due to the thermal sensitivity of these phenolic compounds. An extended duration of extraction at a higher temperature could improve the extraction yield but it could also lead to the decomposition and hydrolytic degradation of some thermo-labile phenolic compounds [69]. Consistent with our results, some previous studies have found also that TPC of the extracts increased with temperature; however, the extraction temperature differently impacted the content of individual polyphenolic compounds in the extracts [70]. For instance, Aaby et al. [47] and other previous studies [71,72] found that flavonols from bilberries are stable during processing while cinnamic acid-containing compounds, and most of all chlorogenic acid, are less stable during bilberry juice processing and bilberry press residue extraction. Aaby et al. [47] also reported that flavonols and cinnamic acid-containing compounds had the highest contribution to total phenolics in the aqueous extracts of bilberry press residue obtained after extraction for 30 min at 22 °C, while the contribution of anthocyanins increased with temperatures up to 60 °C, then declined in the extracts obtained at 100 °C.

Anthocyanins are among the main phenolic fractions in bilberry pomace [32,34,73]. Čanadanović-Brunet et al. [25] identified ten individual anthocyanins in bilberry pomace extract, of which petunidin and cyanidin glycosides were the predominant ones. Paes et al. [74] identified sixteen anthocyanins in the residues of blueberry juice processing and reported that cyanidin 3-*O*-glucoside was the major anthocyanin. In contrast, Klavins et al. [75] found that delphinidin 3-*O*-glucoside was the major anthocyanin in the bilberry press residue extract.

The content of cyanidin 3-glucoside significantly increased in the apple juices after the extraction of the bilberry pomace as the extraction temperature increased from 20 °C to 80 °C. In a study investigating the impact of temperature on both total anthocyanins and total phenolic content, Ju and Howard [76] found an optimal extraction temperature of anthocyanins from dried red grape skin between 80 and 100 °C, and around 120 °C for total phenolic content. In contrast, Cacace and Mazza [77] reported the decline of the anthocyanin content in the TPC of blackcurrant extracts made with aqueous ethanol (from 70% to 54%) as a result of an increase in temperature beyond 45 °C. Aaby et al. [47] also found that the yield of flavonols and cinnamic acid-containing compounds in the aqueous extracts of bilberry pomace increased with increasing extraction temperature. Here, it is worth pointing out that other extraction conditions, such as solvent type, solvent concentration, extraction time and acidity, greatly influence the recovery of phenolic compounds in the extract [78].

The high content of anthocyanins in enriched apple juices is extremely valuable because they impart the characteristic blue–purple color of wild bilberries and improve the juice’s biological activities through their well-known antioxidant, anti-inflammatory, antitumor, neuroprotective and eye function properties [79].

## 4. Conclusions

The present study confirmed the great potential of bilberry pomace as a source of bioactive compounds and showed that extracting bilberry pomace in apple juice contributed to the enrichment of the final beverage in anthocyanins and other phenolic compounds. Response surface methodology combined with the Box-Behnken design were successfully employed to compare and optimize the parameters for TAC, TPC and RSA ultrasonic-assisted extraction. Based both on the observed and predicted responses, the most desirable results for bioactive compounds (TAC and TPC) and antioxidant activity (RSA) in the enriched juices were obtained after 60 min of ultrasound-assisted extraction of 15% bilberry pomace percent at 80 °C temperature. The HPLC results showed that, besides anthocyanins, chlorogenic, gallic, caffeic, 3-hydroxybenzoic, p-coumaric, ellagic and protocatechuic acids, as well as epigallocatechin and catechin, were the main phenolics extracted from bilberry pomace directly in apple juice. Positive strong correlations between TAC, TPC and RSA in the enriched juice samples were determined. Due to the high content of anthocyanins and other polyphenols, the new enriched apple juice is a high nutraceutical and functional beverage, with considerable potential for health-promoting properties and the prevention of chronic and degenerative diseases caused by oxidative stress. Moreover, the valorization of bilberry pomace could protect the environment and positively impact the local industry development.

## Figures and Tables

**Table 1:** Coded and actual values of the process parameters (independent variables) used in the Box–Behnken design.

Independent Variables		Coded Values	
	−1	0	+1
		Actual Values	
*x*_1_: Temperature (degrees Celsius)	20	50	80
*x*_2_: Extraction time (minutes)	30	60	90
*x*_3_: Solid percent (%)	5	10	15

**Table 2:** Response values (mean ± SD, n = 2) of total anthocyanin content, total phenolic content and DPPH radical scavenging activity for actual levels of process parameters (temperature, extraction time and solid percent) in RSM.

Process Parameters (Actual Values)			Total Anthocyanin Content (mg CGE/L)	Total Phenolic Content (mg GAE/L)	DPPH Radical Scavenging Activity (mmol Trolox/L)
Temperature (°C)	Extraction Time (min)	Solid Percent (%)			
80	60	5	90.58 ± 3.08	950.91 ± 34.45	4.33 ± 0.18
20	30	10	70.19 ± 2.55	605.45 ± 24.48	3.89 ± 0.16
20	60	5	41.73 ± 1.67	460.00 ± 20.11	2.72 ± 0.12
50	90	5	84.41 ± 2.98	646.36 ± 28.11	3.78 ± 0.15
80	30	10	180.69 ± 6.68	1150.91 ± 40.09	6.44 ± 0.28
50	30	15	187.80 ± 5.98	1291.82 ± 43.34	6.87 ± 0.28
50	60	10	147.01 ± 6.14	991.82 ± 26.66	5.74 ± 0.24
50	90	15	203.45 ± 8.29	1400.91 ± 56.06	7.26 ± 0.31
80	90	10	174.99 ± 7.41	1273.64 ± 43.33	7.02 ± 0.32
50	30	5	76.83 ± 3.12	628.18 ± 21.98	3.70 ± 0.17
20	90	10	75.88 ± 3.77	950.91 ± 35.56	3.91 ± 0.16
50	60	10	137.53 ± 5.55	991.82 ± 37.56	5.45 ± 0.21
80	60	15	262.73 ± 11.33	1700.91 ± 56.02	8.72 ± 0.38
50	60	10	140.38 ± 4.88	1055.45 ± 39.88	5.58 ± 0.26
20	60	15	98.64 ± 3.48	932.73 ± 36.78	4.95 ± 0.20

**Table 3:** Regression coefficients and analysis of variance (ANOVA) of the predicted quadratic model to the responses for total anthocyanin content, total phenolic content and DPPH radical scavenging activity.

Regression Coefficients	Total Anthocyanin Content	Total Phenolic Content	DPPH Radical Scavenging Activity
*β*_0_	−29.3457 * (0.0000)	−185.631 * (0.0000)	0.840694 * (0.0000)
*β*_1_ (temperature)	1.74674 * (0.0000)	7.87036 * (0.0004)	0.0283056 * (0.0000)
*β*_2_ (extraction time)	0.220444 (0.1495)	6.53422 (0.0679)	−0.00498611 * (0.0150)
*β*_3_ (solid percent)	3.28042 * (0.0000)	35.9472 * (0.0001)	0.24975 * (0.0000)
*β*_12_	−0.00316389 (0.2908)	−0.0618694 (0.2740)	0.000155556 * (0.0434)
*β*_13_	0.192067 * (0.0001)	0.462117 (0.1868)	0.0036 * (0.0001)
*β*_23_	0.01345 (0.4410)	0.151517 (0.6374)	0.000516667 (0.1968)
*β*_11_	−0.0171694 * (0.0016)	0.000843056 (0.9878)	−0.000276389 * (0.0059)
*β*_22_	−0.0008333 (0.7771)	−0.0206236 (0.7103)	−0.0000291667 (0.6486)
*β*_33_	−0.1107 (0.3205)	−0.10605 (0.9574)	−0.00645 * (0.0310)
Lack-of-fit	0.5431	0.0969	0.8956
*R* ^2^	99.781	97.3616	99.861

* *p*-values (shown in parentheses) below 0.05 indicate statistically significant effects at the 95.0% confidence level.

**Table 4:** Pearson correlations (*R*) between total anthocyanin content, total phenolic content and DPPH radical scavenging activity of the enriched apple juice.

	Total Phenolic Content	DPPH Radical Scavenging Activity
Total anthocyanin content	0.950828 * (0.0000)	0.989893 * (0.0000)
Total phenolic content		0.963782 * (0.0000)

* *p*-values (shown in parentheses) below 0.05 indicate statistically significant correlations at the 95.0% confidence level.

**Table 5:** Phenolic compound content (mg/L) determined by HPLC-DAD in apple juice and juices enriched with bioactive compounds through direct ultrasound-assisted extraction from bilberry pomace.

Phenolic Compound	AJ	AJBP20	AJBP50	AJBP80
Gallic acid	23.37 ± 0.47 ^a^	23.77 ± 0.46 ^a^	24.13 ± 0.66 ^a^	25.71 ± 0.55 ^b^
Epigallocatechin	33.30 ± 0.61 ^a^	42.67 ± 0.88 ^b^	43.07 ± 0.73 ^b^	45.02 ± 0.48 ^c^
Catechin	54.59 ± 1.04 ^a^	73.47 ± 1.33 ^b^	78.95 ± 0.97 ^c^	82.55 ± 1.65 ^d^
Chlorogenic acid	136.22 ± 2.11 ^a^	181.42 ± 3.77 ^b^	199.74 ± 3.98 ^c^	186.41 ± 4.66 ^b^
Caffeic acid	4.04 ± 0.17 ^a^	4.64 ± 0.19 ^b^	6.20 ± 0.18 ^c^	6.82 ± 0.24 ^d^
3-Hydroxybenzoic acid	18.66 ± 0.23 ^a^	19.67 ± 0.31 ^b^	21.18 ± 0.27 ^c^	19.96 ± 0.19 ^b^
Cyanidin 3-glucoside	nd	129.29 ± 5.52 ^a^	220.67 ± 6.67 ^b^	316.94 ± 8.78 ^c^
p-Coumaric acid	nd	2.08 ± 0.11 ^b^	1.46 ± 0.06 ^a^	1.39 ± 0.05 ^a^
Ellagic acid	2.75 ± 0.12 ^a^	20.72 ± 0.76 ^b^	25.69 ± 1.11 ^c^	25.17 ± 0.98 ^c^
Ferulic acid	1.98 ± 0.07 ^b^	1.87 ± 0.08 ^ab^	1.95 ± 0.09 ^ab^	1.82 ± 0.06 ^a^
Protocatechuic acid	0.02 ± 0.01 ^a^	7.12 ± 0.28 ^c^	2.68 ± 0.12 ^b^	0.02 ± 0.01 ^a^
Resveratrol	0.45 ± 0.02 ^a^	0.44 ± 0.02 ^a^	0.42 ± 0.03 ^a^	0.53 ± 0.02 ^b^
Quercetin	nd	1.99 ± 0.08 ^b^	1.83 ± 0.09 ^a^	1.78 ± 0.06 ^a^
Kaempferol	9.28 ± 0.32	nd	nd	nd
Total	284.70 ± 5.18 ^a^	511.47 ± 13.79 ^b^	622.34 ± 14.96 ^c^	714.15 ± 17.73 ^d^

Data expressed as mg/L of juice and presented as mean ± standard deviation. nd—not detected; Different superscript letters indicate significant (*p* < 0.05) differences between sample juices. AJ—control apple juice; AJBP20, AJBP50 and AJBP80 are juices obtained through direct ultrasound-assisted extraction of bioactive compounds from 10% bilberry pomace during 30 min at 20 °C, 50 °C and 80 °C, respectively.

## Data Availability

The original contributions presented in this study are included in the article. Further inquiries can be directed to the corresponding author.
